# Neural Network Used for the Fusion of Predictions Obtained by the K-Nearest Neighbors Algorithm Based on Independent Data Sources

**DOI:** 10.3390/e23121568

**Published:** 2021-11-25

**Authors:** Małgorzata Przybyła-Kasperek, Kwabena Frimpong Marfo

**Affiliations:** Institute of Computer Science, Faculty of Science and Technology, University of Silesia in Katowice, Bȩdzińska 39, 41-200 Sosnowiec, Poland; kmarfo@us.edu.pl

**Keywords:** neural network, fusion method, independent data sources, k-nearest neighbors algorithm, dispersed data

## Abstract

The article concerns the problem of classification based on independent data sets—local decision tables. The aim of the paper is to propose a classification model for dispersed data using a modified k-nearest neighbors algorithm and a neural network. A neural network, more specifically a multilayer perceptron, is used to combine the prediction results obtained based on local tables. Prediction results are stored in the measurement level and generated using a modified *k*-nearest neighbors algorithm. The task of neural networks is to combine these results and provide a common prediction. In the article various structures of neural networks (different number of neurons in the hidden layer) are studied and the results are compared with the results generated by other fusion methods, such as the majority voting, the Borda count method, the sum rule, the method that is based on decision templates and the method that is based on theory of evidence. Based on the obtained results, it was found that the neural network always generates unambiguous decisions, which is a great advantage as most of the other fusion methods generate ties. Moreover, if only unambiguous results were considered, the use of a neural network gives much better results than other fusion methods. If we allow ambiguity, some fusion methods are slightly better, but it is the result of this fact that it is possible to generate few decisions for the test object.

## 1. Introduction

The article is devoted to the issue of classification based on dispersed data. More precisely, data collected in many local decision tables, which were provided by independent units, are considered. This approach is considered for example in federated learning [1,2] and fog computing [3] approaches.

The considered approach differs from an ensemble classifiers approach mainly due to the form of data that is considered. In ensemble learning, a set of local tables is generated based on one decision table in a controlled manner. Thus, we can define the form of attributes’ sets and objects’ sets in local tables, and that simplifies a lot. It is completely different in the case of dispersed data. The set of local decision tables is already collected independently, and we have no influence on its form. In addition, sets of attributes in local tables can be quite different, but some elements may be common between sets. A similar property applies to sets of objects. In addition, we cannot expect that we have universal identifiers for objects in all local tables, so it is impossible to verify which objects are common between local tables.

In real applications, very often, data are collected in such a dispersed way. In specialized departments located in various hospitals, various banks, or even on smart phones—information about the user. Each application installed locally can collect a personalized local table. The domain of such dispersed data can be very different. However, the general problem remains the same—how to use dispersed data efficiently considering all inconsistent local tables at the same time. This is very significant and one of the top problems in today’s multi-devices world.

This leads to completely different considerations, causes inconsistencies and conflicts between local tables. In addition, there are very important issues regarding data protection and data privacy [4]. We do not deal with this problem in the publication; however, we follow all the rules by not sharing raw data, only prediction results in central domain.

A common approach to deal with dispersed data is to build a separate local model based on each local table and then combine the local prediction results [5,6,7]. In the stage of combining the prediction results, we can use fusion methods [8] from three different levels (measurement level, rank level and abstract level). Another approach is to build a meta-model that will train on how to generate global results based on predictions obtained from local models.

Fusion methods very often generate ambiguous results in the case of dispersed data (ties occur). The motivation of the study is to propose a model that will generate unambiguous results for dispersed data. We know that neural networks can learn to recognize even multilayer and complex patterns very well. Prediction results generated by local tables (especially from the measurement level) are often contradictory, ambiguous and it is difficult to generate a global decision based on them. Therefore, neural networks seem to be the appropriate approach to working with such data.

This paper proposes the use of a neural network in conjunction with a modified *k*-nearest neighbors algorithm for classification problems based on dispersed data. In the literature, we can find applications of neural networks in the stage of predictions fusion [9,10,11]. These applications are in specialized fields and rely on the use of completely different methods to generate local predictions than those proposed in this paper.

The contribution of this paper is to propose a classification model for dispersed data using a modified *k*-nearest neighbors algorithm and a neural network. The first step in the model is to use the *k*-nearest neighbors algorithm to generate prediction vectors based on local tables. Prediction vectors are defined over the decision classes. Therefore, it was required to modify this algorithm due to the need to generate certainty that the object belongs to each specific decision class. This algorithm ensures that the prediction vector will be determined based on the most relevant objects to the currently considered test object. In the next stage, it is proposed to use a neural network to generate the final decision. The network is trained to make the correct decision based on such prediction vectors. It is not an easy task, because total agreement is very unlikely to be obtained in the prediction vectors. Particular systems of values in vectors may indicate a specific decision value. It may happen that some local tables are better in recognizing objects from a specified subspace. Neural networks seem to be an appropriate model for recognizing all these complex relationships.

The paper is organized as follows. In Section 2, the proposed classification model using dispersed data is described. Each of the stages in the model—generating local predictions and combining local predictions is presented in a separate subsection. The algorithm’s description and the discussion about the computational complexity are given. Then, a graphical presentation of the method and an overall overview are presented. In the last subsection, other fusion methods that are used in this article as the baseline method are also discussed. Section 3 addresses the data sets that were used and presents the conducted experiments and discussion on obtained results. Section 4 is on conclusions and future research plans.

## 2. Materials and Methods

This section describes the classification model for dispersed data available in many local decision tables. This classification process consists of two stages:

- At the first stage, a model is built based on each local decision table. Using a modified *k*-nearest neighbors algorithm, predictions from the measurement level are designated.
- At the second stage, a neural network is used to aggregate the predicted results to determine the final classification.

### 2.1. The First Stage in a Dispersed Classification Model

We assume that a set of decision tables is given. The tables were collected independently by separate units. Based on each table, a classifier is built. We assume that a set of decision tables $D_{ag}=\left(U_{ag},A_{ag},d\right),\;ag\in Ag$ from one discipline is available, where $U_{ag}$ is the universe, a set of objects; $A_{ag}$ is a set of conditional attributes; *d* is a decision attribute. Decision tables are collected independently, so both sets of objects and sets of attributes can have any form. They can have common elements between tables, but do not have to. The only condition that must be met by local tables is collecting data from one discipline. Formally, this is satisfied by the assumption that the same decision attribute is present in all tables. Aggregation for such tables is difficult and can generate inconsistencies, therefore aggregation is not performed, but predictions are made separately based on each table.

Ag is a set of classifiers and an identifier ag∈Ag is a classifier that is built based on a decision table $D_{ag}$. In general, any classifier may be used for this purpose. In this study, the modified *k*-nearest neighbors classifier with the Gower similarity measure was used. For each local table and for each test object *x*, a probability vector over decision classes (denoted by $\mu_{ag}\left(x\right)$) is designated. The dimension of vectors $\mu_{ag}\left(x\right)=\left[\mu_{ag,1}\left(x\right),\ldots ,\mu_{ag,c}\left(x\right)\right]$ is equal to the number of decision classes $c=card\{V^{d}\}$, where $V^{d}$ is a set of decision attribute values from all decision tables and $card\{V^{d}\}$ is the cardinality of this set. Each coefficient $\mu_{ag,j}\left(x\right)$ is determined using the *k*-nearest neighbors of the test object *x* belonging to a given decision class *j* and decision table $D_{ag}$. The Gower similarity measure [12] is used because it allows the analysis of data with different types of attributes and data with missing values. At the end of this stage, we obtain for each test object a set of vectors over the decision classes in a cardinality equal to the number of local tables.

In this way, we obtain local predictions, but not the final global decision. The k-nearest neighbors method is used for the computation of local predictions, because this method is not computationally complex and easily scalable even for large and multidimensional data. Moreover, in the *k*-nearest neighbors algorithm, predictions rely strictly on the relevant objects from the data sets. Thanks to this, we obtain diversified classifiers for dispersed data (as local tables are independent), which, as we know from the literature [13], is important for an ensemble of classifiers. In addition, the *k*-nearest neighbors method has already been used in previous studies for dispersed data [14,15] and has produced good results. To generate the final global decision, we propose training a neural network in making the final decision using the probability vectors generated based on local tables.

The pseudo-code of the algorithm that generates predictions based on local tables is given in Algorithm 1.

The computational complexity of the above algorithm is rather small and equal to $O(card\left\{Ag\right\}\times \max_{ag\in Ag}card\left\{U_{ag}\right\}\times card\left\{U_{test}\right\})$.

### 2.2. The Second Stage in a Dispersed Classification Model

As the results of the previous stage, we obtain a set of vectors over the decision classes $\mu_{ag}\left(x\right),x\in U_{test},ag\in Ag$. However, we do not have a final decision for test objects. To determine a global decision for the test object *x*, we must fuse the vectors $\mu_{ag}\left(x\right),ag\in Ag$. We propose the use of a neural network for this purpose. More formally, a multilayer perceptron is applied. We use one network structure. This network consists of three layers: an input, a hidden layer and an output layer. The input of the neural network are the values of vectors generated in the previous step of classification. The number of neurons in the input layer is equal to the number of local decision tables multiplied by the number of decision classes $card\left\{Ag\right\}\times card\left\{V^{d}\right\}$. Thus, the greater the data dispersion, the greater the complexity. The number of neurons in the output layer is equal to the number of decision classes. Each of the neurons determines the probability with which the test object belong to a given decision class.
**Algorithm 1** Pseudo-code of algorithm generating predictions based on local tables**Input:** A set of local decision tables $D_{ag}=\left(U_{ag},A_{ag},d\right),ag\in Ag$; test set—a decision table $D_{test}=\left(U_{test},A_{test},d_{test}\right)$, $A_{test}={⋃}_{ag\in Ag}A_{ag}$**Output:** A set of vectors over decision classes $\mu_{ag}\left(x\right),ag\in Ag,x\in U_{test}$. foreach $x\in U_{test}$
        foreach ag∈Ag                  Calculate the value of the Gower similarity measure for each object in the set $U_{ag}$                  and the test object *x* (using only the attributes from the set $A_{ag}$).                  For each *j*-th decision class, find the *k* nearest neighbors objects to *x* from *j*-th                  decision class of the decision table $D_{ag}$.                  $\mu_{ag,j}\left(x\right)$ is equal to the mean of similarity of the *k* nearest neighbors objects from                  the *j*-th decision class of the decision table $D_{ag}$.        end foreachend foreach


In this study, different numbers of neurons in the hidden layer are analyzed. Such a number should depend on the number of neurons in the input layer, and this is dependent on the data. Therefore, the following values will be analyzed: {1,3,4,4.25,4.5,4.75,5}× the number of neurons in the input layer. For the hidden layer, the ReLU (Rectified Linear Unit) activation function is used, as it is the most popular activation function and gives very good results [16]. For the output layer, the SoftMax activation function is used, which is recommended when we deal with a multi-class problem [17]. In this paper, data sets containing from four to 19 decision classes are analyzed. The neural network is trained using the backpropagation method. A gradient descent method, with an adaptive step size is used in the backpropagation method. It is known that the SoftMax layer give good results with the Adam optimizer [18]. The Adam optimizer was proposed in [19] and is one of the most popular adaptive step size methods. From [20] we know that the categorical cross-entropy loss gives best results with SoftMax layer. That is why the Adam optimizer and the categorical cross-entropy loss function are used in the study.

The code that is used defines a neural network using Keras library in Python.

The pseudo-code of the algorithm that defines a neural network with one hidden layer is given in Listing 1.

Listing 1 **Neural network with one hidden layer.**

**def** neural_model(inputDim : **int**, neurons : **int**):
       model = Sequential()
       model . add(Dense(neurons, input_dim=inputDim,
                                         activation=’relu’))
       model . add(Dense(classes, activation=’softmax’))
       model . **compile** (loss=’categorical_crossentropy’,
                                     optimizer=’adam’, metrics=[’accuracy’])
**return**  model

The neural network is trained based on the test set. It should be emphasized that the objects from the training set are not used for this purpose. The objects from the training set are used to determine the probability vectors, i.e., the *k*-nearest neighbors classifier is used for the training set. Thus, to study the classification quality of neural network, the 10-fold cross-validation method is used for test set. Thus, each time nine folds from the test set are used to train the neural network, while the last fold is used to analyze the classification quality.

**Figure 1:**
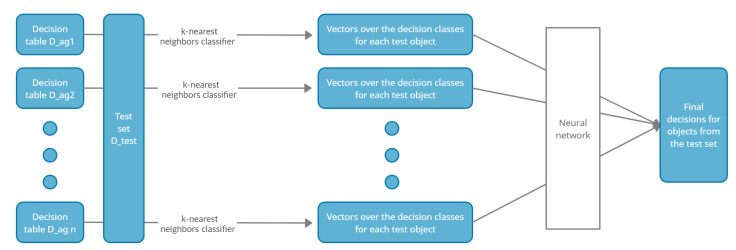
Stages in the dispersed classification model.

Figure 1 presents the described above stages in a dispersed classification model.

In the proposed model, two main algorithms are used. A modified *k*-nearest neighbors algorithm and a neural network. The first one has low computational complexity and classifies test objects based on dispersed local tables. Due to the dispersion of data and the differences in the attributes sets in local tables, it is not possible to generate an aggregated result using this algorithm. Therefore, it is necessary to use a different method of designating the global decision. For this purpose, a neural network is used. The training process of the neural network is a complex process. However, it should be remembered that it is only implemented once. The process of test objects’ classification in the neural network is a task with very low computational complexity. It consists only of performing arithmetic operations in the number depending on the number of layers in the network and the number of neurons in each layer. The great advantage of neural network used as a fusion method is obtaining unambiguous results. This approach does not generate ties—one decision class is generated always.

### 2.3. Other Fusion Methods

The results obtained using the proposed method are compared with the results generated by other fusion methods known from the literature. These methods can be used to aggregate the predictions in the form of probability vectors. Thus, a modified *k*-nearest neighbors algorithm is used as described above. However, instead of a neural network, the results are aggregated using the fusion method.

For this purpose, five methods were selected. The chosen methods are from different measurement groups, they are characterized by completely different criteria and have different degrees of complexity. Three of the methods are simple and very popular. The other two methods are much more computationally complex and sophisticated.

The first method from the measurement level is the sum rule. The sum rule consists of the designation of the sum of the probability values assigned to one decision class by each of local tables. The set of decisions that have the maximum of these sums is the final decision

$$\arg\max_{j\in \{1,\ldots ,c\}}\left\{\sum_{ag\in Ag}\mu_{ag,j}\left(x\right)\right\}.$$

The next fusion method, the Borda count method, belongs to the rank level, which means that the ranks vectors are generated based on the probability vectors. Ranks are assigned within vectors in such a way that decision classes that have greater probability also have higher rank. The method consists of designating the sum of the number of classes ranked below the given class by each local decision table. Thus, the value is determined for each decision class *j*$\sum_{ag\in Ag}\left(card\left\{V^{d}\right\}-r_{ag,j}\left(x\right)\right),$ where $r_{ag,j}\left(x\right)$ is the rank assigned based on the probability value $\mu_{ag,j}\left(x\right)$. The set of classes that have the maximum value of the Borda count is the final decision.

The third fusion method that is used is the majority voting method and belongs to the abstract level. In this method, each local table gives one vote for each decision that has the maximum probability in the probability vectors. Final decisions are those which received the maximum number of votes.

The next two methods are much more complex and sophisticated, both require a training stage.

The method that is based on decision templates belongs to the measurement level and was proposed in the paper [21]. The method uses decision profile, i.e., the matrix with dimension number of local tables × number of decision classes. The rows of the matrix are the probability vectors from the measurement level generated based on local tables. This is the way of presenting the local tables outputs for any object. For each class, the decision templates are defined based on the decision profiles that are constructed for the objects from the training set. The decision template $DT_{j}$ for class *j* is the average of the decision profiles of the objects of the training set in class *j*. These decision templates can be seen as patterns for decision classes and the process of determining them is the training process of the method. When a test object is considered, first a decision profile for the object is determined, then, the similarity between the decision profile for the test object and the decision template for each class is calculated. For this purpose, one of the distance measures is used. The Euclidean distance, the Hamming distance, the Jaccard similarity or the Symmetric difference are usually applied. The set of classes that have the maximum value of similarity is the final decision. In this article, the Hamming distance is used, because based on previous experience [22] it produces the best results.

The method that is based on the theory of evidence belongs to the measurement level and was proposed in the paper [23]. In this method as in the previous method, the decision templates for decision classes $DT_{j},j\in \left\{1,\ldots ,c\right\}$ are designated based on the training set. The decision templates and the decision profile for the test object are compared using the Dempster-Shafer theory and the belief is calculated. The following steps are performed in the Dempster-Shafer algorithm:

1. Let $DT_{j}\left(m,\cdot \right)$ denote the *m*-th row of the decision template for class *j* and $DP_{m,\cdot }\left(x\right)$ denote the *m*-th row of the decision profile for the object *x*. The proximity between the prediction calculated based on the *m*-th local table $DP_{m,\cdot }\left(x\right)$ and the *m*-th row of the decision template for every class j∈{1,…,c} and for each local table *m* is calculated
  $$\phi_{j,m}\left(x\right)=\frac{{\left(1+{∥DT_{j}\left(m,\cdot \right)-DP_{m,\cdot }\left(x\right)∥}^{2}\right)}^{-1}}{\sum_{k=1}^{c}{\left(1+{∥DT_{k}\left(m,\cdot \right)-DP_{m,\cdot }\left(x\right)∥}^{2}\right)}^{-1}}$$
  where $∥\cdot ∥$ is the norm. The Euclidean norm was applied in this study.
2. For every class j∈{1,…,c} and for each local table *m* the following belief degrees are calculated
  $$Bel_{j}\left(DP_{m,\cdot }\left(x\right)\right)=\frac{\phi_{j,m}\left(x\right)\prod_{k\neq j}\left(1-\phi_{k,m}\left(x\right)\right)}{1-\phi_{j,m}\left(x\right)\left[1-\prod_{k\neq j}\left(1-\phi_{k,m}\left(x\right)\right)\right]}.$$
3. The Dempster-Shafer membership degrees for every class j∈{1,…,c} are calculated
  $$\mu_{j}\left(x\right)=K\prod_{m}Bel_{j}\left(DP_{m,\cdot }\left(x\right)\right)$$
  where *K* is a constant that ensures that $\mu_{j}\left(x\right)\leq 1$.

The set of classes that have the maximum value of the Dempster-Shafer membership degrees is the final decision.

All the above-mentioned fusion methods can generate ties. In such a situation, instead of one final decision, a set of decisions is generated. In this article, these ties are not solved. In the next section, two measures of classification quality are analyzed to compare the ambiguity of results.

## 3. Results

The experiments were conducted with the data taken from the UC Irvine Machine Learning Repository and one artificially generated data. Each data available in the repository is stored in a single table. Before the experiments were performed, the data were dispersed. For this reason, not all data sets could be used for analysis. An important issue is the presence of many conditional attributes in the data. Moreover, the proposed model uses the Gower measure, which is dedicated to data with different types of attributes (qualitative, quantitative, binary). Therefore, it is important that the attributes in the data set are of different types. The existence of multi-decision classes in a data set is also an important matter, as some of the fusion methods used for comparison may generate ties. Ties in the case of a small number of decision classes (in extreme cases two decision classes) are not acceptable.

Three data sets meeting the above conditions were selected for the analysis: the Lypmhography, the Vehicle Silhouettes and the Soybean (Large) data sets. For the Soybean data set, the two independent data sets: a test set and a training set are available in the repository. The Vehicle and the Lymphography data sets were randomly divided into two disjoint subsets, the training set (70% of objects) and the test set (30% of objects). This was done to apply the same testing strategy for all analyzed data sets (train and test method).

The artificial data were generated using the Weka software [24]. For this purpose, the function RandomRBF was used. This function, at first, randomly generates centers for each decision class. Then to each center, a weight is randomly assigned and a central point per attribute, and a standard deviation. The new object is generated as follows. The center is selected—according to the weights. Attribute values are randomly generated and offset from the center. Then the vector is scaled so that its length equals a value sampled randomly from the Gaussian distribution of the center. The decision class is assigned based on the center. The following settings were used:

- Number of numerical conditional attributes—30
- Number of objects—399
- Number of decision classes—7
- Number of centroids (To make the set more difficult, several centroids were used to generate objects from one decision class)—50
- Seed for generating random numbers—1

Thus, unbalanced data were obtained, the numbers of objects in individual decision classes are as follows: 21,36,31,57,58,107,89. The data set was randomly divided into two disjoint subsets, the training set (299 objects) and the test set (100 objects) in a stratified mode. Additionally, noise has been added to each attribute in the training set. For this purpose, the values generated from the normal distribution with an average of 0 and a standard deviation of 0.3 were used and added to each value in the training set.

For dispersed data, the cross-validation method is too complex. Moreover, the cross-validation method would be difficult to apply because the test set should contain objects that have defined values on all the conditional attributes that belong to local tables. In addition, this restricts, and even makes it impossible to draw independently from local tables. Data characteristics are given in Table 1.

The training sets of the above-mentioned data sets were dispersed. To check for different degrees of dispersion for each data set, five different dispersed versions with 3, 5, 7, 9 and 11 local tables were prepared. Conditional attributes for local tables were selected randomly, but each local table contained only a small subset of the full set of attributes. Certain attributes were common to several local tables. The decision attribute was included in each of the tables. The full set of objects is also stored in each of the local tables, but without identifiers. This reflects the real situation where we cannot identify the objects between the tables.

The quality of classification was evaluated based on the test set. Three measures were analyzed. The first measure is the estimator of classification error *e*. It is a fraction of the total number of objects in the test set that were classified incorrectly. An object is considered to be correctly classified when its correct decision class belongs to the generated decision set. The second measure is the estimator of classification ambiguity error $e_{ONE}$. It is also a fraction of the total number of objects in the test set that were classified incorrectly. However, this time an object is considered to be correctly classified when only one correct decision class was generated. More strictly, this measure does not accept ambiguity. The third measure is the average number of generated decisions sets $\bar{d}$. The third measure allows an assessment of how often and how numerous are the draws generated by the dispersed classification model and the fusion methods.

It should be noted once again that to use the neural network, a 10-fold cross-validation was used on the test set, i.e., the neural network was trained 10 times with 9 folds and tested on one remaining fold. In addition, each test was performed three times to ensure that the results were reliable and not distorted by the influence of randomness. The results for the neural network approach that are given below are the average of the obtained results.

The experiments were carried out according to the following scheme:

- Generating vectors of predictions based on local tables using the *k*-nearest neighbors classifier. For each data set, three different values of the *k* parameter were tested, namely k∈{1,5,10}. One parameter value was selected for each data set that produced the best overall results. For the Vehicle Silhouettes data set—k=5, for the Lymphography data set—k=10, for the Soybean data set—k=1, for the artificial data set—k=1 were selected.
- Generating a global decision using a neural network with one hidden layer and different number of neurons in the hidden layer. For each data set, the following number of neurons in the hidden layer were tested: {1,3,4,4.25,4.5,4.75,5}× the number of neurons in the input layer. Different number of neurons in the hidden layer was also checked. However, it was noticed that the accuracy of the respective models improves as the number of neurons in the hidden layer increases, but significant improvement declines around 5× the number of neurons in the input layer. The number of neurons in the input layer depends on the number of local tables. Thus, the more dispersed data we have, the more complex the structure of the neural network is.
- Generating a global decision using one of the fusion methods: the sum rule, the Borda count, the majority vote, the method that is based on decision templates and the method that is based on theory of evidence.

Comparison of experimental results was made in terms of:

- The quality of classification for different numbers of neurons in the hidden layer;
- The quality of classification of the proposed dispersed classification model vs. other fusion methods (the sum rule, the Borda count, the majority vote, the method that is based on decision templates and the method that is based on theory of evidence).

### 3.1. Comparison of Experimental Results for Different Numbers of Neurons in the Hidden Layer

Table 2 presents average classification error *e*, average classification ambiguity error $e_{ONE}$ and the average number of generated decisions set $\bar{d}$ for all dispersed data sets and the neural network approach with different number of neurons in the hidden layer. Using 10-fold cross-validation method, experiments were repeated 3 times. The minimal mean errors have been marked in bold. A very important thing that should be noticed is that all generated results are unambiguous. Always a single decision class is generated. So, for the proposed model we have the property $e=e_{ONE}$ and $\bar{d}=1$.

Based on results from Table 2, the calculated means and the individual results for the dispersed data sets, it can be seen that the best results are obtained for the number of neurons in the hidden layer of approximately equal 4× the number of neurons in the input layer.

The Friedman’s test was performed. All results (the number of neurons in the hidden layer: {1,3,4,4.25,4.5,4.75,5}× the number of neurons in the input layer) were selected—each number of neurons as a separate group, the test confirmed that differences among the classification error in these seven groups are significant, with a level of p=0.002599. Then, to determine the pairs of groups between which statistically significant differences occur, the Wilcoxon each pair test for dependent groups were performed. The test showed that there is significant difference with p<0.05 between

- Group 1 (1 × #Input) and four other groups (Group 2—3 × #Input, Group 3—4 × #Input, Group 4—4.25 × #Input and Group 5—4.5 × #Input),
- Group 2 (3 × #Input) and three other groups (Group 3—4 × #Input, Group 4—4.25 × #Input and Group 5—4.5 × #Input),
- Group 3 (4 × #Input) and Group 7—5 × #Input,
- Group 4 (4.25 × #Input) and Group 7—5 × #Input.

**Figure 2:**
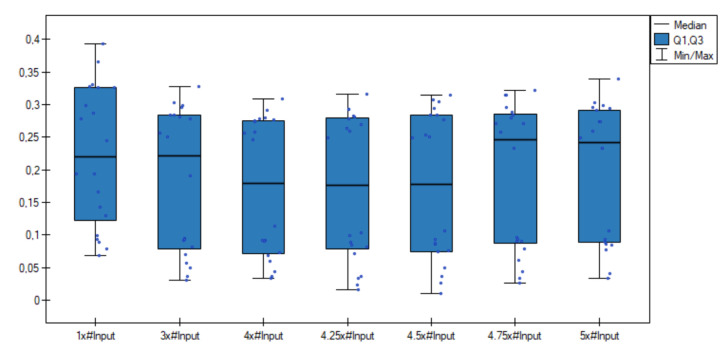
Box-plot chart with (Median, the first quartile—Q1, the third quartile—Q3) the value of classification error *e* for the neural network with different numbers of neurons in the hidden layer.

Additionally, comparative box-plot chart for the values of the classification error was created (Figure 2). As can be observed, distributions of the classification error values in groups are quite different—especially better results are visible for the numbers of neurons in the hidden layer equal to 4 × #Input and 4.25 × #Input.

### 3.2. Comparison of Experimental Results for the Proposed Dispersed Classification Model versus Other Fusion Methods

Table 3 presents classification error *e*, classification ambiguity error $e_{ONE}$ and the average number of generated decisions set $\bar{d}$ for all dispersed data sets and fusion methods: the sum rule, the Borda count, the majority voting, the method that is based on decision templates and the method that is based on theory of evidence. These results were obtained using train and test method. The tests were performed on the test sets on which the neural network was trained and tested at an earlier stage of the experiments. As before, the following *k* values were used in the modified *k* nearest neighbors algorithm: the Vehicle Silhouettes data set—k=5, for the Lymphography data set—k=10, for the Soybean data set—k=1 and for the artificial data set—k=1. The tests were performed only once as these fusion methods are deterministic. As can be seen, virtually all results are ambiguous. Only for the method that is based on decision templates and the method that is based on theory of evidence always one decision class is generated. Additionally for the Vehicle data set and the sum rule one decision class is generated.

If we compare the ambiguous results (values *e*) generated by the fusion methods and those obtained with the use of the neural network, then it can be concluded that the results generated by the neural network are worse, in most cases not much (for example the Soybean data set). However, such a comparison is not objective, as this improvement is obtained by allowing ambiguity. It is only in the case of the artificial data set that the neural network approach produces better results. This is due to the fact that the artificial data have numerical attributes and the fusion methods do not generate as much ambiguity.

Table 4 presents the $e_{ONE}$ values obtained with the use of neural network (best for all tested numbers of neurons in the hidden layer) and the considered fusion methods. The smallest ambiguity errors have been marked in bold.

Based on results from Table 4, it can be seen that the best results are obtained for the neural network approach. Moreover, it can also be seen that for more dispersed data (on a larger number of local tables) the proposed model still generates good results. On the other hand, other fusion methods generate increasingly worse results in the case of large dispersion of data.

The Friedman’s test was performed. All results were selected—each fusion method as a separate group, the test confirmed that differences among the classification ambiguity error in these four groups are significant, with a level of p<0.000001. Then, to determine the pairs of groups between which statistically significant differences occur, the Wilcoxon each pair test for dependent groups were performed. The test showed that there is significant difference with p<0.05 for all pairs except between:

- Group 3 (Borda count) and two other groups (Group 5—Decision templates and Group 6—Theory of evidence),
- Group 4 (Majority voting) and two other groups (Group 5—Decision templates and Group 6—Theory of evidence),
- Group 5 (Decision templates) and Group 6 (Theory of evidence).

**Figure 3:**
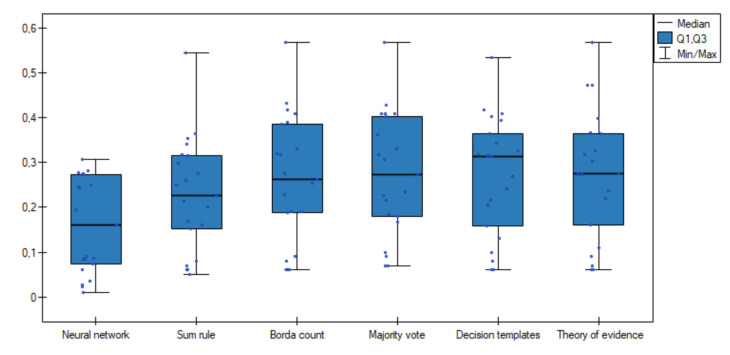
Box-plot chart with (Median, the first quartile—Q1, the third quartile—Q3) the value of classification ambiguity error *e* for the neural network and other fusion methods.

Additionally, comparative box-plot chart for the values of the classification ambiguity error was created (Figure 3). As can be observed, distributions of the classification ambiguity error values in groups are completely different—especially better results were obtained for the proposed neural network method.

All experiments were performed on a portable computer with the following technical specifications:

- Intel i7-8565U CPU,
- 16 GB of RAM memory,
- Ubuntu 18.04.5 LTS operating system.

The algorithm has been implemented in Python and all the data-related calculations have been saved in a text document.

The main advantages and limitations of the proposed approach using the *k*-nearest neighbors algorithm and the neural network are listed below.

The main advantages are:

- The proposed model always generates unambiguous results for both numerical and qualitative data set.
- When the unambiguous results are compared, a much better quality of classification are obtained using the proposed model than using the other fusion methods: the majority voting, the Borda count method, the sum rule, the method that is based on decision templates and the method that is based on theory of evidence.
- The deviation of the results obtained by the proposed model is much smaller compared to the deviation of the results obtained for other fusion methods, which can be seen on Figure 3.
- Based on the performed experiments, it can be concluded that in most cases the number of neurons in the hidden layer equal to 4× the number of neurons in the input layer generates the best results. This parameter value can be adopted for related future work without the need to use a complex analysis of the network structure for each new data set separately.

The main limitations are:

- Unfortunately, neural networks do not provide a clear and human-interpretable formula for global decision making. A human readable principle, a rule for combining decisions or some pattern, is not generated.
- To apply the proposed model, we must have quite a large test set available to train the neural network. In the case of other fusion methods, such a condition does not have to be met. They can generate a global decision even in the case of only one test object being available.

## 4. Conclusions

In this article, a new model using a modified *k*-nearest neighbors algorithm in conjunction with a neural network to generate decisions based on dispersed data—available from independent data sources is proposed. The paper presents a comparison of the results obtained with the use of the proposed model in comparison with other fusion methods known from the literature. This comparison was made using the data sets from the repository and an artificially generated data set. Both numerical and qualitative data were checked. Moreover, various degrees of dispersion into local tables of the analyzed data were analyzed. In the article, various structures of the neural network in terms of the number of neurons in the hidden layer were studied.

The obtained results show that the proposed model always generates unambiguous decisions. This is a big advantage of this approach as fusion methods very often generate ties, especially for qualitative data. In addition, it can be seen that the proposed model can deal equally well with data that is finely dispersed (11 local decision tables) and those that are less dispersed (three local decision tables). This statement is not true for other fusion methods which definitely produce worse results for highly dispersed data.

The comparison of the ambiguity classification error clearly showed that the proposed model generates better results than the other considered fusion methods. When accepting ties, fusion methods especially the Borda count method and the majority voting produce better results, but this is the result of allowing for ambiguity. Moreover, the results obtained with the use of the proposed approach are characterized by a much smaller deviation.

In the paper, the optimal number of neurons in the hidden layer of the neural network was determined. It is similar for all analyzed data sets; therefore, it can be used in related future works for new data sets.

The main limitations of the proposed approach are the need to provide a quite large test set to train the neural network. The model does not generate clear and interpretable rules according to which the global decision is determined.

In future research, it is planned to use different activation functions in the neural network. It is also planned to allow for ambiguity by applying multiple logistic regressions function in the output layer of neural network.

It would be very interesting to compare the performance of the proposed model with neural networks for different parameters of the data sets. It is planned to perform comparative experiments in future research:

- generating artificial data with a different number of conditional attributes, e.g., from 20 to 100 in steps of 20; the influence of the dimensionality of the data set;
- generating artificial data of different noise intensity; use the normal distribution with the standard deviation from 0.1 to 0.5 in steps of 0.1; the influence of the noise intensity;
- generating artificial data with a different number of outliers.

The presented studies focus on showing that the proposed approach has potential and will be developed in the future. 

## Figures and Tables

**Table 1:** Data set characteristics.

Data Set	# The Training Set	# The Test Set	# Conditional Attributes	# Decision Classes
Vehicle Silhouettes	592	254	18	4
Lymphography	104	44	18	4
Soybean	307	376	35	19
Artificial Data	299	100	30	7

**Table 2:** Results of classification error *e*, classification ambiguity error $e_{ONE}$ and the average number of generated decisions $\bar{d}$ for the dispersed system with neural network. Designation #Input is used for the number of neurons in the input layer.

Data Set	No. on Local Tables	No. of Neurons in Hidden Layer						
		1 × #Input	3 × #Input	4 × #Input	4.25 × #Input	4.5 × #Input	4.75 × #Input	5 × #Input
		$e=e_{\mathrm{ONE}}$/$\bar{d}$	$e=e_{\mathrm{ONE}}$/$\bar{d}$	$e=e_{\mathrm{ONE}}$/$\bar{d}$	$e=e_{\mathrm{ONE}}$/$\bar{d}$	$e=e_{\mathrm{ONE}}$/$\bar{d}$	$e=e_{\mathrm{ONE}}$/$\bar{d}$	$e=e_{\mathrm{ONE}}$/$\bar{d}$
Lypmho	3	0.286/1	0.281/1	**0.246**/1	0.259/1	0.251/1	0.288/1	0.273/1
graphy	5	**0.194**/1	0.251/1	0.258/1	0.264/1	0.253/1	0.258/1	0.259/1
	7	0.326/1	0.328/1	**0.308**/1	0.316/1	0.314/1	0.321/1	0.339/1
	9	0.278/1	0.256/1	0.256/1	**0.249**/1	**0.249**/1	0.271/1	**0.249**/1
	11	**0.244**/1	0.278/1	0.276/1	0.269/1	0.276/1	0.271/1	0.294/1
Vehicle	3	0.365/1	0.296/1	0.279/1	0.283/1	0.284/1	0.096/1	0.089/1
	5	0.331/1	0.284/1	**0.278**/1	**0.278**/1	0.294/1	0.296/1	0.291/1
	7	0.326/1	0.299/1	0.291/1	**0.281**/1	0.304/1	0.284/1	0.298/1
	9	0.298/1	0.284/1	**0.275**/1	0.293/1	0.307/1	0.314/1	0.295/1
	11	0.327/1	0.303/1	**0.274**/1	0.293/1	0.284/1	0.315/1	0.302/1
Soybean	3	0.093/1	0.092/1	0.091/1	**0.084**/1	0.085/1	0.096/1	0.089/1
	5	0.088/1	0.094/1	0.091/1	0.099/1	0.093/1	0.093/1	**0.085**/1
	7	0.099/1	0.093/1	0.090/1	0.089/1	**0.087**/1	0.090/1	0.093/1
	9	0.078/1	0.081/1	**0.073**/1	0.081/1	0.075/1	0.079/1	0.084/1
	11	0.068/1	0.069/1	0.068/1	0.071/1	0.074/1	**0.061**/1	0.077/1
Artificial	3	0.393/1	0.190/1	0.113/1	0.103/1	0.106/1	**0.090**/1	0.106/1
Data	5	0.193/1	0.056/1	0.060/1	**0.023**/1	0.026/1	0.033/1	0.233/1
	7	0.166/1	0.030/1	0.033/1	0.016/1	**0.010**/1	0.233/1	0.233/1
	9	0.143/1	0.036/1	0.036/1	0.033/1	0.036/1	**0.026**/1	0.033/1
	11	0.130/1	0.050/1	0.043/1	**0.036**/1	0.050/1	0.043/1	0.040/1
average *e*		0.221	0.183	0.172	0.171	0.173	0.187	0.197

**Table 3:** Results of classification error *e*, classification ambiguity error $e_{ONE}$ and the average number of generated decisions $\bar{d}$ for fusion methods—the sum rule, the Borda count, the majority voting, the method that is based on decision templates and the method that is based on theory of evidence.

Data Set	No. of Local Tables	Sum Rule *e*/$e_{\mathrm{ONE}}$/$\bar{d}$	Borda Count *e*/$e_{\mathrm{ONE}}$/$\bar{d}$	Majority Vote *e*/$e_{\mathrm{ONE}}$/$\bar{d}$	Decision Templates *e*/$e_{\mathrm{ONE}}$/$\bar{d}$	Theory of Evidence *e*/$e_{\mathrm{ONE}}$/$\bar{d}$
Lypmho	3	0.250/0.250/1	0.159/0.386/1.227	0.136/0.409/1.273	0.159/0.159/1	0.273/0.273/1
graphy	5	0.273/0.318/1.045	0.205/0.318/1.114	0.205/0.318/1.114	0.318/0.318/1	0.318/0.318/1
	7	0.273/0.341/1.068	0.205/0.432/1.227	0.205/0.409/1.250	0.364/0.364/1	0.364/0.364/1
	9	0.205/0.364/1.159	0.182/0.409/1.227	0.182/0.409/1.227	0.409/0.409/1	0.364/0.364/1
	11	0.273/0.545/1.273	0.205/0.568/1.364	0.205/0.568/1.364	0.205/0.205/1	0.273/0.273/1
Vehicle	3	0.260/0.260/1	0.256/0.276/1.035	0.232/0.307/1.165	0.315/0.315/1	0.366/0.366/1
	5	0.299/0.299/1	0.280/0.319/1.067	0.264/0.362/1.150	0.417/0.417/1	0.472/0.472/1
	7	0.276/0.276/1	0.291/0.331/1.055	0.283/0.331/1.079	0.394/0.394/1	0.398/0.398/1
	9	0.354/0.354/1	0.339/0.390/1.063	0.299/0.402/1.146	0.402/0.402/1	0.472/0.472/1
	11	0.315/0.315/1	0.358/0.417/1.067	0.311/0.429/1.161	0.535/0.535/1	0.567/0.567/1
Soybean	3	0.117/0.170/1.085	0.120/0.189/1.106	0.082/0.215/1.247	0.314/0.314/1	0.303/0.303/1
	5	0.101/0.152/1.077	0.117/0.191/1.106	0.082/0.184/1.199	0.343/0.343/1	0.327/0.327/1
	7	0.088/0.202/1.149	0.109/0.253/1.189	0.072/0.234/1.295	0.327/0.327/1	0.237/0.237/1
	9	0.072/0.160/1.106	0.104/0.191/1.101	0.061/0.168/1.146	0.242/0.242/1	0.221/0.221/1
	11	0.088/0.213/1.157	0.088/0.229/1.184	0.090/0.226/1.181	0.215/0.215/1	0.160/0.160/1
Artificial	3	0.050/0.050/1	0.060/0.060/1.020	0.030/0.070/1.080	0.060/0.060/1	0.060/0.060/1
Data	5	0.060/0.060/1	0.050/0.060/1.030	0.060/0.070/1.040	0.060/0.060/1	0.060/0.060/1
	7	0.060/0.060/1	0.060/0.060/1	0.040/0.090/1.080	0.080/0.080/1	0.070/0.070/1
	9	0.070/0.070/1	0.070/0.080/1.010	0.060/0.100/1.050	0.100/0.100/1	0.090/0.090/1
	11	0.080/0.080/1	0.060/0.090/1.030	0.140/0.180/1.100	0.130/0.130/1	0.110/0.110/1

**Table 4:** Classification ambiguity error $e_{ONE}$ for neural network and other fusion methods.

Data Set	No. on Local Tables	Neural Network $e_{\mathrm{ONE}}$	Sum Rule $e_{\mathrm{ONE}}$	Borda Count $e_{\mathrm{ONE}}$	Majority Vote $e_{\mathrm{ONE}}$	Decision Templates $e_{\mathrm{ONE}}$	Theory of Evidence $e_{\mathrm{ONE}}$
Lypmho	3	0.246	0.250	0.386	0.409	**0.159**	0.273
graphy	5	**0.194**	0.318	0.318	0.318	0.318	0.318
	7	**0.308**	0.341	0.432	0.409	0.364	0.364
	9	**0.249**	0.364	0.409	0.409	0.409	0.364
	11	0.244	0.545	0.568	0.568	**0.205**	0.273
Vehicle	3	0.274	**0.260**	0.276	0.307	0.315	0.366
	5	**0.278**	0.299	0.319	0.362	0.417	0.472
	7	0.281	**0.276**	0.331	0.331	0.394	0.398
	9	**0.275**	0.354	0.390	0.402	0.402	0.472
	11	**0.274**	0.315	0.417	0.429	0.535	0.567
Soybean	3	**0.084**	0.170	0.189	0.215	0.314	0.303
	5	**0.085**	0.152	0.191	0.184	0.343	0.327
	7	**0.087**	0.202	0.253	0.234	0.327	0.237
	9	**0.073**	0.160	0.191	0.168	0.242	0.221
	11	**0.061**	0.213	0.229	0.226	0.215	0.160
Artificial	3	0.090	**0.050**	0.060	0.070	0.060	0.060
Data	5	**0.023**	0.060	0.060	0.070	0.060	0.060
	7	**0.010**	0.060	0.060	0.090	0.080	0.070
	9	**0.026**	0.070	0.080	0.100	0.100	0.090
	11	**0.036**	0.080	0.090	0.180	0.130	0.110
average $e_{ONE}$		0.160	0.227	0.262	0.274	0.269	0.275

## Data Availability

Publicly available data sets were analyzed in this study. These data can be found here: [25]. One data set has been artificially generated description of the way is presented in the paper.
